# Validation of the Khorana Score to Assess Venous Thromboembolism and Its Association with Mortality in Cancer Patients: A Retrospective Community-based Observational Experience

**DOI:** 10.7759/cureus.7883

**Published:** 2020-04-29

**Authors:** Gulrayz Ahmed, Hira Gulrayz Nasir, Kathryn Hall, Lisa Weissmann

**Affiliations:** 1 Internal Medicine, Medical College of Wisconsin, Milwaukee, USA; 2 Internal Medicine, Dow University of Health Sciences, Karachi, PAK; 3 Preventive Medicine, Brigham and Women's Hospital, Harvard Medical School, Boston, USA; 4 Internal Medicine, Mount Auburn Hospital, Harvard Medical School, Cambridge, USA

**Keywords:** khorana score, thrombosis, venothromboembolism, deep venous thrombosis, pulmonary embolism, cancer

## Abstract

Introduction: Khorana score (KS) stratifies patients into low, intermediate, and high risk groups for venous thromboembolism (VTE). We examined the generalizability of the KS to risk of VTE and association with mortality.

Methods: A retrospective cohort study was conducted at Mount Auburn Hospital, Cambridge, Massachusetts. Patients aged 18 years or older undergoing chemotherapy were included. All patients were evaluated for a six-month period. Primary study endpoints were VTE or mortality.

Results: Some 277 participants were included with a mean age of 63.95 (standard deviation, SD ± 12.47). The incidence proportion was 6.13% and a total of 17 VTE events were reported over a 2.5-year period. Compared to those with a low KS (0), those with a high KS (3 or above) had 6.4 times (p=0.032) while with an intermediate KS (1-2) had 2.6 times the odds of having a VTE event (p=0.22).

Those who had a VTE had 4.03 times the odds of death compared to those who did not have a VTE (p=0.006). Compared to those with a low KS, those with a high KS had 5.7 times (p=0.02) the odds of six-month mortality and 5.04 odds (p=0.001) of mortality at any time.

Conclusion: High KS was associated with increased odds of VTE and mortality in our study.

## Introduction

Venous thromboembolism (VTE) is one of the major causes of morbidity, and the second most frequent cause of death in a cancer population [1-2]. Cancer patients are at a more than four times greater risk of thrombosis as compared to the general population [3]. Cancers of the colon, pancreas, and brain pose the greatest risk. For example pancreatic cancer is reported to be associated with 22%-36% risk of having a VTE [4]. Patients with both cancer and VTE have an up to eight-fold greater risk of death as a result of a thrombotic event compared to patients without cancer [5]. While there is significant evidence supporting the relationship between cancer and thrombosis in the literature, the pathophysiology remains unclear [5].

Additional factors also contribute to a greater risk of VTE occurrence among cancer patients, such as increased BMI, increased platelet count, increased leukocyte count, and diminished hemoglobin levels [6]. Also of significance, many cancer therapies, including chemotherapy, surgery, and hormonal therapy, have been shown to cause a dramatically greater incidence of VTE in patients [5]. Currently there exist no recommendations for universal anticoagulation of cancer patients at high risk for VTE, likely due to increased risk of bleeding and lack of published literature in this regard [1]. Therefore, a system to stratify patients at risk for thrombotic events could identify those subsets of patients who might derive the best risk to benefit ratio. In 2008, Khorana et al. proposed an objective risk model that could be used to stratify patients into low, intermediate, and high risk groups for VTE [7]. This scoring system, termed the Khorana score (KS), is based on easily available objective data and has been studied in academic medical teaching centers to assess the risk of VTE [8]. There is strong evidence that this scoring system is highly predictive of the risk for thrombotic events in cancer patients [9]. Moreover, there is also some evidence that a high KS is associated with increased mortality [9-11].

To date the KS and other scoring systems for high risk of VTE have been assessed and studied at major research centers, but the majority of the US population seeks care in community setting hospitals. The prevalence and risk for thrombotic events may be different in this group of patients as in general they may be older, less mobile, and have higher rates of co-morbidities than patients seeking care at a major research center. In this study we assessed the generalizability of the KS to risk of VTE in a community hospital and determined association with mortality.

## Materials and methods

A retrospective cohort study was conducted at the outpatient Hematology and Oncology clinic of Mount Auburn Hospital, Cambridge, Massachusetts. Patients 18 years or older undergoing chemotherapy for a newly diagnosed cancer or new chemotherapy treatment after disease progression/re-occurrence with at least three months of lapsed time from prior chemotherapy were included in this study. The Khorana predictive model for cancer and chemotherapy-associated VTE was used to evaluate the risk of developing a VTE [7]. Exclusion criteria were patients -- with insufficient documentation in the electronic medical records, enrolled in clinical trials, with recent chemotherapy treatment within three months, who had surgery within the past two weeks, who had a past medical history of VTE, and patients on continuous anticoagulation.

Patients were evaluated for a six-month period beginning from January 2016 to December 2017 for VTE events. De-identified data collected during the study included patient characteristics (age, sex, KS), past medical history (chronic co-morbidities, current aspirin use, prior VTEs), characteristics of cancer (location, type, stage, prior chemotherapy, last round of chemotherapy). The outcomes we recorded were incident VTE, pulmonary embolism (PE), and death. Ultrasound dopplers or CT (PE Protocol) was used to confirm a diagnosis of VTE.

Data were collected and analyzed using STATA (Stata Statistical Software: Release 16. College Station, TX). Simple logistic regression, chi-square, and frequencies were used for analysis. Primary study endpoints included VTE or mortality at six months. We also continued to follow the patients and their mortality was documented anytime during the subsequent 2.5-year study period. This study was approved by the Internal Review Board at Mount Auburn Hospital.

## Results

Demographic characteristics

A total of 339 patients were screened, of which 277 were found to meet the eligibility criteria. The mean age was 63.95 (SD ± 12.47) with a median of 65 years (25-89). Detailed demographics are presented in Table 1.

**Table 1:** Demographics.

	n	%
Total n	277	
Age, mean (SD)	63.95 (SD ±12.47)	
Sex		
Female	174	62.8
Male	103	37.2
Cancer Location		
Bladder	11	4.0
Breast	64	23.1
Chronic lymphocytic leukemia	2	0.7
Colon cancer	18	6.5
Gynecologic	32	11.6
Hodgkin's lymphoma	5	1.8
Lung	48	17.3
Multiple myeloma	6	2.2
Non-Hodgkin's lymphoma	20	7.2
Others	43	15.5
Pancreas	7	2.5
Prostate	8	2.9
Rectal	5	1.8
Stomach	5	1.8
Testicular	3	1.1
Cancer Stages		
1	30	10.8
2	64	23.1
3	66	23.8
4	106	38.3
Unstageable	11	4.0

A total of 17 VTE events (6.1%) were observed during the study period, of which four were PE and the remaining events were classified as venous embolism of extremities or abdominal vasculature. Stage 1 cancer patients had no VTE events while stages 2-4 were associated with 3, 4, and 10 events respectively. Further findings are discussed in Table 2.

**Table 2:** Findings KS, Khorana score; ASA, acetyl salicylic acid; VTE, venous thromboembolism

	n	%
Total n	277	
KS		
High (>=3)	36	13.0
Intermediate (1-2)	160	57.8
Low (0)	81	29.2
Aspirin (ASA) use		
Yes	213	76.9
No	64	23.1
Platelet Count		
< 350	83	30.0
> or = 350	194	70.0
VTE Events	17	6.1
Deaths	78	27.8

Of the 62 patients excluded from the study, five had a VTE event in less than three months, 26 had prior chemotherapy in the last three months, while 35 had a known comorbidity for which they were already on anticoagulation.

An increase in KS was associated with VTE

Compared to those with a low KS (0), those with a high KS (3 or above) were more likely to have a VTE event [odds ratio, OR = 6.4 (95% confidence interval, CI 1.17-34.58), p=0.032]. The difference between low and intermediate KS (1-2) was not significant [OR=2.6 (95% CI 0.56-12.31), p=0.22]. Patients with metastatic cancer had higher likelihood of having a VTE event than those without metastatic cancer, though this was not statistically significant [OR= 2.28 (95% CI 0.84-6.18), p=0.11]. The risk of VTE tended to be higher with aspirin use compared to patients not using aspirin, though this difference did not reach statistical significance [OR = 2.81 (95% CI 0.98-8.04), p=0.06].

VTE and KS were associated with death

During the entire study period of 2.5 years, VTE was associated with an increased risk of death [OR=4.03 (95% CI 1.48-11.02), p=0.006]. Compared to those with a low KS, those with a high KS were at greater risk of death in the first six months of follow-up [OR=5.7 (95% Cl 1.32-24.66), p=0.02], and mortality at any time in the study [OR=2.54 (95% Cl 1.87-13.54), p=0.001]. Intermediate KS also showed increased mortality which was statistically significant during the overall study period [OR= 2.54 (95% Cl 1.26-5.11), p=0.009] but not significant at a six month interval [OR=2.49 (95% Cl 0.7-8.94), p=0.161]. Compared to those with a low KS, those with a high KS had 5.04 times the odds of mortality, adjusting for cancer stage (metastatic vs. nonmetastatic) [(95% Cl 1.87-13.54), p=0.001]. Compared to those with a low KS, those with an intermediate KS had 2.54 times the odds of mortality, adjusting for cancer stage [(95% Cl 1.19-5.42), p = 0.016]. Findings remained unchanged even after adjusting for age and sex.

## Discussion

Our study reports that even in a community hospital, high KS is associated with increased odds of VTE as well as mortality. The occurrence of VTE, either in the form of deep venous thrombosis (DVT) or PE, leads to significant morbidity and mortality in cancer patients. Of the 500,000 new cases of VTE diagnosed annually in the United States, approximately 20% are associated with malignancy [12]. VTE among cancer patients is multifactorial and various factors including tumor type, stage of disease, chemotherapy/hormonal therapy, surgical interventions, age, immobilization, prior VTE, and other factors interplay their part [1]. In addition, it has been postulated that endothelial damage, alterations in the blood flow, and presence of pro-coagulants all cause abnormal clotting and could contribute to the development of VTE in cancer patients due to the tumor itself or to anticancer therapy [2].

In the 277 patients we studied, the cumulative incidence of VTE was 6.13%, which is consistent with other published studies [1, 12-13]. The KS was developed and validated on a total of 4066 patients with various cancer types to identify patients at sufficiently high risk for VTE. The goal of the KS was to determine which patients required thromboprophylaxis to improve morbidity and mortality [9]. Expanded uses for the score have emerged over the past decade to improve delivery of cancer therapy, cancer-related outcomes, quality of life, and use of healthcare resources.

While multiple studies have validated the KS as a predictive tool for chemotherapy-associated VTE, there are some studies that say otherwise [9, 14-18]. Our study validated KS in a community setting with a predominant elderly population and showed that high KS as well as intermediate KS was associated with increased odds of VTE but only high KS when compared to low KS was statistically significant (p=0.032). The Vienna Cancer and Thrombosis study -- a prospective observational cohort study of 819 patients initiating outpatient cancer treatment (including surgery and radiotherapy in addition to chemotherapy) -- found that the cumulative probability of developing VTE at six months increased significantly with a patient’s KS. In a retrospective chart review of 112 patients with solid tumors or malignant lymphoma who had undergone chemotherapy within the previous two years, the incidence of VTE also increased with KS [12].

Consistent with other published literature, our study reported that patients with VTE, compared to no VTE, were at significantly increased risk of mortality. Kuderer et al. in their study reported that VTE was associated with significantly worse prognosis than patients without VTE [10]. Based on our analysis, people with high KS also had increased mortality at six month interval as well as in the overall study period. When this finding was adjusted for metastatic vs. nonmetastatic disease, it remained significant showing that KS is an independent indicator irrespective of cancer stage [OR 2.57 (95% Cl 1.16-5.76], p=0.02]. In the Vienna Cancer and Thrombosis study a KS score of >3 when compared to KS of 0 had fourfold higher mortality risk after adjusting for age, sex, and incident VTE. However, the link between higher KS and an increased rate of death was independent of VTE occurrence, suggesting that the KS identified susceptibilities in addition to VTE that can cause death [6]. This finding was replicated in a prospective study in 719 patients with lung cancer, where patients in the high risk KS category were at increased risk of death [17].

Our observation that a majority of our study patients were on aspirin (76.9%), but there was a trend towards an increased risk of having VTE with aspirin is hypothesis-generating and warrants further exploration in other cohorts. This observation is in line with the American Society of Clinical Oncology’s current guidelines against using aspirin for thromboprophylaxis [19]. Despite the prevalence of aspirin use, 44.5% in some studies, several other studies found no benefit of aspirin use on reducing incidence of VTE events in cancer patients [20-21].

Though this study gives an insight into the real-world experience of using KS, it also has several limitations. This is a small retrospective single center study. We were limited by the small sample size which may account for the large CI and lack of precision in the OR estimates and reliability of the results. The effect of specific chemotherapies was not evaluated or controlled for. Our institute caters to higher proportion of Caucasian elderly population and findings might not be applicable to racially diverse younger population.

## Conclusions

In this study high KS was associated with increased odds of VTE and mortality after adjusting for cancer stage. High KS and intermediate KS were also statistically associated with increased mortality at any time. Aspirin use showed no protective effect and was associated with increased odds of VTE events though not statistically significant.
